# Topologically Structured Light in Nanophotonics

**DOI:** 10.1002/nap2.70187

**Published:** 2026-07-01

**Authors:** Yijie Shen, Qiwen Zhan, Harald Giessen

**Affiliations:** ^1^ School of Physical and Mathematical Sciences & School of Electrical and Electronic Engineering Nanyang Technological University Singapore; ^2^ Department of Electronic and Information Engineering, School of Engineering Westlake University Hangzhou Zhejiang China; ^3^ 4th Physics Institute and Research Center SCoPE, University of Stuttgart Stuttgart Germany

In recent years, the exploration of topology in optical fields has grown into an exceptionally topical and exponentially expanding research domain. Originating from the sophisticated tailoring of phase, amplitude, and polarization profiles, structured light has extended its touch from classical light shaping to advanced photon‐matter interaction, from transverse pattern to higher dimensional complexity [1, 2, 3]. Recently, structured light has been married with topological structures and nanophotonics, offering an unique playground where fundamental physics thrives and unexpected propagation regimes emerge with remarkable robustness against defects or disorders [4, 5, 6, 7].

It is within this exciting context that we celebrate the successful completion of this special collection on “Topologically Structured Light in Nanophotonics”. Spanning across core themes from optical vortex and vector beam engineering to functional metasurfaces, optical topological quasiparticles, and, for the first time, interaction of structured light with nanostructured matter, this collection encapsulates the state‐of‐the‐art milestones in the field and outlines inspiring future trajectories for next‐generation classical and quantum information technologies. Altogether, this special collection comprises 25 high‐quality articles that vividly reflect the diversity, dynamism, and visual impact of the field. The published works present a compelling blend of comprehensive reviews by pioneers and cutting‐edge original research pushing the boundaries of physical science and device engineering.

To navigate the rich tapestry of research gathered here, the contributions can be broadly categorized into four defining themes that currently drive the field. The first theme of the special collection is on optical topological quasiparticle textures. Real‐space particle‐like topological textures of light, such as optical skyrmions, merons, and hopfions, have attracted intense attention for their particle‐like properties and robust topologies [4, 5, 6, 7]. This special collection highlights significant breakthroughs in the creation, tracking, and characterization of these exotic quasiparticles. Firstly, Harald Giessen's group reports on tunable skyrmion, meron, and skyrmion bag textures in surface phonon polariton lattices [8], whereas Filippo Cardano and colleagues' group explores skyrmionic polarization textures in structured dielectric planar media [9]. Furthermore, Yijie Shen's group introduces spatiotemporal skyrmionic structures of a new class of free space‐time higher‐order wave packets [10]. Natalia Litchinitser's group demonstrates methods of structuring skyrmionic polarization textures in space‐time [11]. Jin Liu's group presents key advancements in generating bright single‐photon skyrmion sources using bullseye cavities [12]. Qirong Xiao's group generates optical skyrmions with longitudinal dynamics evolution from metasurfaces [13]. Fanfei Meng and co‐workers provide insights into the near‐field characterization and manipulation of topological spin structures of light [14]. Then, Qiwen Zhan's group demonstrates the topological decomposition and transformation of spin quasicrystal textures [15]. Xiaocong Yuan's group further uncovers the hidden spin‐momentum locking and topological defects on hyperbolic metamaterials [16], collectively opening new frontiers for localized metrology and topological protection diagnostics.

The second theme discusses recently advanced meta‐platforms for light‐shaping toolkits. The modern toolkit for customizing topological light has fundamentally shifted toward flat optics, integrated devices, and structured materials. Metasurfaces leveraging geometric and propagation phases have become indispensable for steering spatial and temporal degrees of freedom.

Under this research direction, Xinyuan Fang's group presents the nanograting arrays enabled application of high‐capacity multiview display with large viewing angle via orbital angular momentum encoding [17]. Jingbo Sun and coauthors demonstrate innovative on‐chip Spherulite platforms for generating white vortex light with a broad band [18]. Additionally, contributions from Robert Fickler's team demonstrate nonlinear meta‐platforms for ultrafast rewritable structured light holography with extreme spatial precision [19]. This section is further enriched by cutting‐edge research from Fatemeh Davoodi, whose work highlights topological photonic rings as an advanced platform for chiral coherent dynamics from bound states to quantum spin models [20].

The third theme focuses on the novel photonic vortex modes, angular momentum lasers, and their diagnostics. Efficiently sorting, generating, and propagating orbital angular momentum (OAM) fields remain central to advancing high‐dimensional optical processing. In a standout cover feature, Shiyao Fu and co‐workers outline the state‐of‐the‐art regarding solid‐state vortex laser sources at eye‐safe bands [21].

Moving from generation to modal manipulation, Xinzhong Li and coauthors report an elegant solution for fractional orbital angular momentum sorting using a novel method of wind spiral transformations and show its advantage in high‐capacity communications [22]. Mark Dennis and Kerr Maxwell introduce Euler‐top Gaussian modes, charting structured beams derived from quadratic angular momentum dynamics [23]. To unlock new diagnostics and mapping paradigms, Miguel A. Alonso demonstrates the quantum geometric phase of the commensurate bichromatic polarization and astigmatic cavity systems [24], whereas Thomas Juffmann's group applies the concept of quantum Fisher information to enhance localization precision in coherent scattering microscopy using structured light [25]. Finally, Christina Jörg's group perspectives the frontiers of harnessing higher‐order vortex modes in photonic simulators [26].

In the fourth theme, nontrivial effects of structured light in complex media and applications were discussed. Beyond fundamental generation, harnessing structured wavefronts to drive applications across scattering channels, telecom fibers, and biological tissues represents a crucial technological milestone.

In this context, Ram Nandan Kumar and coauthors demonstrate the harvesting of the spin–orbit interaction of light to generate helicity‐dependent complex rotational motion in optically trapped mesoscopic matter [27]. Sonja Franke‐Arnold's group presents a mechanism of atomic state interferometry using complex vector light interaction [28]. Addressing propagation challenges, Igor Meglinski's group evaluates orbital angular momentum in biomedical applications, navigating complex environments while preserving phase integrity [29]. Takashige Omatsu's group reports the demonstration of surface relief formation by irradiating material with complex multivortex modes [30]. Asma Fallah and Eileen Otte interact vortex structured beams with nanoparticles to drive and control multipolar modes [31]. Finally, Noah Apostolico from Harald Giessen's group demonstrated the first interaction of structured light with structured matter [32].

Although this special collection offers a comprehensive snapshot of contemporary achievements, the rapid pace of this field promises even more dazzling horizons ahead. An exciting direction that remains to be further charted lies at the interface of non‐Hermitian topological optics, quantum entangled structured states, and ultra‐high‐power spatiotemporal light–matter interactions in extreme regimes. We are entirely confident that the continuous refinement of our capability to sculpt the topological features of light will significantly enrich the photonics arsenal, fueling future breakthroughs across 6G networks, quantum computing, spectroscopy, and biophotonics.

As Guest Editors, we would like to express our profound gratitude to all the authors for contributing their finest work to this project, and to the reviewers for their rigorous evaluation that sustained the highest academic standards. Finally, our sincere thanks go to the journal's editorial team, particularly Dr. Nadezda Panarina and Dr. Tara Dorrian as well as the original initiative by Dr. Dennis Couwenberg, for their invaluable support, coordination, and dedication that made this beautiful collection possible.

## Author Contributions

**Yijie Shen:** conceptualization, writing – review and editing. **Qiwen Zhan:** conceptualization. **Harald Giessen:** conceptualization.

## Funding

The authors have nothing to report.

## Data Availability

Data sharing is not applicable to this article as no datasets were generated or analyzed during the current study.
